# miRNA biomarkers to predict risk of primary non-function of fatty allografts and drug induced acute liver failures

**DOI:** 10.1007/s11010-024-05129-3

**Published:** 2024-10-18

**Authors:** Juliette Schönberg, Jürgen Borlak

**Affiliations:** Hannover Medical School, Centre for Pharmacology and Toxicology, Carl-Neuberg-Str.1, 30625 Hannover, Germany

**Keywords:** Liver failure, Ischemia injury, Non-coding RNA

## Abstract

Primary non-function (PNF) of an allograft defines an irreversible graft failure and although rare, constitutes a life-threatening condition that requires high-urgency re-transplantation. Equally, drug induced acute liver failures (ALF) are seldom but the rapid loss of hepatic function may require orthotropic liver transplantation (OLT). Recently, we reported the development of a rodent PNF-disease model of fatty allografts and showed that a dysfunctional Cori and Krebs cycle and inhibition of lactate transporters constitute a mechanism of PNF. Based on findings from the rat PNF-disease model, we selected 15 miRNA-biomarker candidates for clinical validation and performed RT-qPCRs in well-documented PNF cases following OLT of fatty allografts. To assess specificity and selectivity, we compared their regulation in pre- and intraoperative liver biopsies and pre- and post-operative blood samples of patients undergoing elective hepatobiliary surgery. Additionally, we assessed their regulation in drug induced ALF. We confirmed clinical relevance for 11 PNF-associated miRNAs and found expression of miRNA-27b-3p, miRNA-122-3p, miRNA-125a-5p, miRNA-125b-5p and miRNA-192-5p to correlate with the hepatic steatosis grades. Furthermore, we demonstrate selectivity and specificity for the biomarker candidates with opposite regulation of let-7b-5p, miRNA-122-5p, miRNA-125b-5p and miRNA-194-5p in blood samples of patients following successful OLTs and/or liver resection. Moreover, by considering findings from 21 independent ALF-studies, we observed nine PNF-associated miRNAs regulated in common. We report miRNAs highly regulated in PNF and ALF, and their common regulation in different diseases broadens the perspective as biomarker candidates. Our study warrants independent confirmation in randomized clinical trials.

**Graphical abstract:**

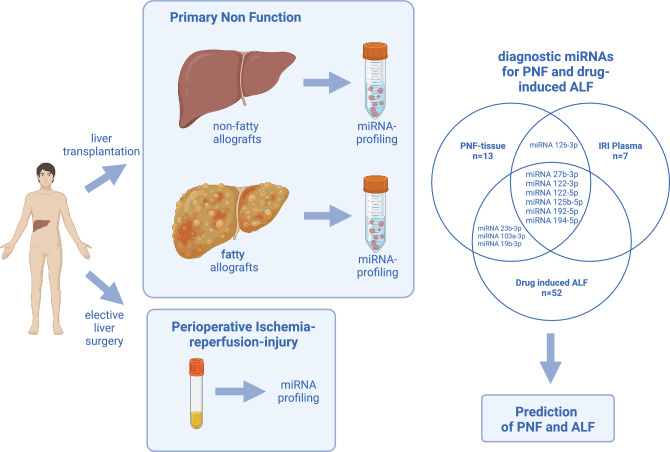

Created in BioRender. Schönberg, J. (2024) BioRender.com/e21p058

**Supplementary Information:**

The online version contains supplementary material available at 10.1007/s11010-024-05129-3.

## Background

Primary non-function (PNF) defines an irreversible graft failure without reasonable surgical or immunological causes [1, 2]. It is a rare, but life-threatening condition and typically requires urgent orthotopic liver re-transplantation (re-OLT).

To better understand causes of PNF, we evaluated the reason, frequency and survival of re-OLT cases among 1,205 orthotopic liver transplantation (OLT) which had been performed at our institution [3]. We considered a wide range of donor and recipient related clinical data. Overwhelmingly, fatty allografts were the main cause for PNF and associated with excessive mortality after re-OLT. Although cold-ischemia and reperfusion injury potentially contributed to the risk of PNF, the Cox proportional-hazard regression analysis defined fatty liver allografts as the sole factor and was independently associated with worse outcome after re-OLT [3].

Importantly, Metabolic Dysfunction-Associated Steatotic Liver Disease (MASLD) has reached pandemic proportions and some estimates suggest a prevalence of up to 90% in clinical obese, 70% in the overweight and about 25% in the general population [4, 5]. Histologically, the presence of macrovesicular lipid droplets in > 5% of hepatocytes defines hepatic steatosis. Typically, pathologists distinguish between micro- and macrovesicular steatosis, and the occurrence of inflammation leading to Metabolic Dysfunction-Associated Steatohepatitis (MASH) and different grades of fibrosis [6, 7]. Although liver biopsies are the golden standard in the diagnosis of MASLD [8], their use in a transplant setting is constrained for a number of reasons. First, biopsies harbor inherent risks of organ damage. Second, it is difficult to obtain specialized histological stains within the time frame of < 12 h between organ procurement and OLT. Third, the procurement of donor organs and the subsequent transplantation frequently involve different hospitals, and the logistics of obtaining a pathology assessment in time can be demanding. Notwithstanding, liver biopsies are obtained from marginal organs with potentially suspicious macroscopic findings [9].

Given the significant shortage of high-quality donor allografts, the need arises to extend the criteria for inclusion of donors (ECD). The outcome of ECD liver transplantation has been the subject of independent reviews [10, 11]. There is clear evidence for fatty liver allografts to be a major risk factor for PNF [3, 12, 13], and the question of how much fat is tolerable in allografts is the subject of a controversial debate.

We recently reported an animal model to investigate mechanisms of fatty liver induced PNF [14] and found hepatic steatosis induced by a methionine choline deficient diet (MCD) to aggravate liver injury induced by the ischemia/reperfusion (IR) injury following OLT. Additionally, fatty allografts suffer from a dysfunctional TCA cycle with major implications for the metabolic competence of the liver. This includes a significant decline in energy/ATP production.

Additionally, drug induced acute liver failure (ALF) is defined by the rapid loss of hepatic functions without prior evidence of chronic liver disease. Estimates based on the United Network for Organ Sharing (UNOS) liver transplant database suggested that about 15% of liver transplants are due to drug hepatotoxicity of which acetaminophen overdose is a common cause [15, 16]. Note, ALF can be self-limiting but may progress with complete loss of hepatic function. Consequently, predicting patients which likely develop a life-threatening situation, and therefore require OLT, is an important unmet medical need.

To identify biomarkers predictive of outcome, we performed genome wide scans in a PNF-disease model of fatty allografts [14]. The significantly regulated miRNAs have an established role in the regulation of hepatic lipid metabolism, injury and liver regeneration as well as programmed cell death and are mechanistically linked to PNF.

In general, miRNAs are small noncoding RNAs (21–23nt) and confer translational repression [17, 18]. Their role as transcriptional regulators in liver diseases is a hot topic [19–21], and miRNAs are of critical importance in the regulation of hepatic lipid synthesis and metabolism [22]. Correspondingly, their contribution to the pathophysiology of fatty liver disease is the subject of intense research [23, 24], and miRNA biomarkers carry the potential to assist in an evaluation of the quality of donor allografts [25].

Based on findings from the PNF animal disease model, we questioned the clinical relevance of regulated miRNAs and given its unpredictable nature, we evaluated PNF-associated miRNAs in formalin-fixed and paraffin-embedded archived tissue (FFPE) of clinical cases and compared the results to histologically normal liver resection material of patients undergoing elective surgery. Furthermore, to demonstrate selectivity, we compared their regulation in blood and liver tissue samples before, intra- and post-surgery for up to 3 days.

Overall, we report a translational study, aimed at defining the clinical relevance of miRNA biomarker candidates derived from an animal model of PNF, and additionally questioned their diagnostic utility in ALF.

## Material and methods

### Sample collection

The basic patient characteristics are given in supplementary Table S1.

## PNF and control liver resection material

We obtained formalin-fixed and paraffin-embedded tissue (FFPE) blocks from the Pathology Institute of Hannover Medical School and assessed liver section material from 29 individual PNF cases and 11 controls. The controls are FFPE tissue blocks of histologically confirmed non-tumor (R0) resection material of patients diagnosed with liver metastasis. The PNF cases were mostly analyzed in duplicate samples and consisted of N = 22 fatty liver allografts and N = 7 non-fatty liver cases as determined by histopathology. The blocks were sectioned to 5 µm thickness, and 10 sections of each block were combined for RNA analysis.

The mean age of patients diagnosed with PNF and colorectal liver metastasis (CLM) patients was 48 and 65 years (supplementary Table S1) and 69 and 91%, respectively were males. Because of the gender disproportionate distributions, we investigated sex-dependent regulation of miRNAs, and as shown in supplementary Figure S1 none were sex-related.

## Liver biopsies from transplant patients and elective surgeries

We obtained biopsies from N = 7 donor livers prior to OLT to enable an assessment of miRNA regulation following ischemia–reperfusion injury (IR injury) and its associated oxidative stress. Additionally, we obtained intraoperative (R0) resection biopsies from N = 10 patients following hilus occlusion of non-tumor material from patients diagnosed with CLM (N = 5) and cholangiocarcinoma (CCC, N = 5). The shock frozen biopsies enabled an assessment of cold- (organ storage, OLT) and warm-ischemia (Hilus occlusion during liver resection) and served as a surrogate endpoint to investigate the effects of organ storage and ischemia injury on the regulation of PNF associated miRNAs. All tissue samples were shock frozen and stored at -80 °C to await further miRNA analysis.

## Plasma samples

Matched blood samples of OLT patients (N = 7) were collected in EDTA tubes about 1 h before surgery (t = -1 h) and on day 1, 2 and 3 post-surgery. Additionally, blood samples of N = 7 patients undergoing elective liver surgery were obtained. As detailed above this group of patients served as an additional control to mimic reperfusion injury after opening of the intra-operative hilus occlusion. The sampling schedule was the same as for liver transplant patients, and the blood samples were centrifuged for 10 min at 2,000 rpm, and the resultant plasma was stored at -80 °C. None of the blood samples were hemolytic.

## RNA isolation FFPE tissue blocks

On a rotary microtome, we prepared a total of 10 sections of 5 µm thickness from single tissue blocks and transferred the material into safe lock tubes. We extracted miRNA with the miRNeasy FFPE Kit according to the manufacturer’s instructions *(Qiagen, Hilden, Germany),* and the sections were treated with 320 µl deparaffinization solution *(Qiagen)* and incubated at 56 °C for 3 min. Next, we added 240 µl of PKD buffer, i.e. a buffer required for proteinase K digestion, vortexed the sample followed by centrifugation at 11,000 g for 1 min. Thereafter, we pipetted 10 µl of proteinase K reagent into the lower (clear) phase of the solution and incubated the sample at 56 °C on a Biometra TS1 Thermo Shaker *(Analytik Jena AG, Jena, Germany)* for 15 min followed by a second incubation step at 80 °C for another 15 min. We transferred the lower, colorless phase into a safe lock tube and stored the sample on ice for 3 min and subsequently centrifuged at 20,000 g in a Thermo Scientific Multifuge X1R *(Thermo Fisher Scientific, Massachusetts, USA)* for 15 min. Once again, we transferred the supernatant into a safe lock tube and added the DNase booster buffer equivalent to 1/10th of the volume in addition to 10 µl DNase I stock solution. The samples were incubated at room temperature for 15 min followed by an addition of 500 µl RBC buffer and vortexed to mix the lysate. Thereafter, we added 1,750 µl of EtOH (100%), vortexed the sample and transferred 700 µl portions to the Rneasy MinElute spin columns. This was followed by the sequential elution with the RPE buffer according to the manufacturer’s recommendations. The eluates were discarded and the Rneasy MinElute spin columns were dried by centrifugation at 14,000 rpm. Finally, the spin columns were conditioned with 20 µl Rnase free water and centrifuged at 14,000 rpm for 1 min. We determined the RNA concentration by measuring the absorbance at 260 nm with the Beckman coulter DU 730 Life Science UV/VIS Spectrophotometer *(Beckman Coulter, California, USA)*. The RNA concentrations ranged from 28.16 ng/µl to 1801.85 ng/µl. We calculated the ratio of 260 nm/280 nm and 260 nm/230 nm absorption to obtain information about the purity of the RNA. Occasionally we performed RNA gel electrophoresis to examine the quality of the isolated RNA and the ribosomal bands.

## Plasma RNA isolation

We isolated total RNA with the miRNeasy serum/plasma kit *(Qiagen, Hilden, Germany)* according to the manufacturer’s recommendations. Briefly, we added 200 µl plasma to 1,000 µl Qiazol and vortexed the sample for 60 s. The samples were incubated at room temperature for another 5 min. Next, we added 3.5 µl miRNeasy serum/plasma spike-in control working solution (= 5.6 × 10^8^ copies) and 200 µl chloroform *(AppliChem, Darmstadt, Germany)* and kept the sample at room temperature for 3 min. Subsequently, we centrifuged the samples at 12,000 g for 15 min and pipetted the upper phase, which contains RNA, into a new tube. We determined the volume (typically 700 µl) and added 100% EtOH at 1.5-fold excess of the initial volume and vortexed the sample. Then, 700 µl portions were applied onto the Rneasy MinElute spin columns followed by an elution step with 700 µl RWT buffer, 500 µl RPE buffer and 500 µl of 80% EtOH. Finally, we centrifuged the spin columns at full speed (~ 12,000 rpm) for 5 min and eluted RNA with 14 µl Rnase free water.

We added a spike-in control, i.e. Cel-miR-39-3p (*Qiagen)* to plasma samples to control the efficiency of the miRNA extraction. The amounts of RNA in plasma were very low, and therefore we could not measure RNA concentrations spectrophotometrically. Instead, we used 1.5 µl of the original RNA extract (see above) for reverse transcription. We prepared a standard curve by blotting different concentrations or copy numbers of the spike-in control and the associated CT-values generated by real-time PCR. We calculated a linear regression and obtained the following equation of the calibration curve: Y = − 3.391*X +48.3

Based on the constructed calibration curve the recovery and therefore efficiency of the extraction could be determined. The data are given as % recovery.

## RNA isolation from resection material

We isolated total RNA from liver resection material of patients undergoing elective hepatobiliary surgery and biopsies taken from donor liver transplants (supplementary Table S1, demographics) with the miRNeasy mini kit *(Qiagen, Hilden, Germany)* according to the manufacturer’s recommendations. The resection material was immediately shock frozen. Typically, we used 20–25 mg frozen tissue from each patient and transferred the material into a vial for further processing. We added 700 µl Qiazol, and the tissue was disintegrated with an Ultra-turrax t10 basic disperser tool *(IKA, Staufen im Breisgau, Germany).* Subsequently, we added 140 µl chloroform *(AppliChem, Darmstadt, Germany)* and kept the sample at room temperature for 3 min. Next, the samples were centrifuged at 12,000 g for 15 min and we pipetted the upper phase, which contains the RNA, into a new tube. We determined the volume (typically 350 µl) and added 100% EtOH at 1.5-fold excess of the initial volume. We vortexed the sample and applied 700 µl portions onto the Rneasy MinElute spin columns followed by elution steps with 350 µl RWT buffer, 500 µl RPE buffer and 500 µl of RPE according to the manufacturer’s recommendations. We centrifuged the spin columns at full speed (~ 12,000 rpm) for 2 min and eluted RNA with 30 µl Rnase free water. We determined the RNA concentrations spectrophotometrically by measuring the absorbance at 260 nm with the Beckman coulter DU 730 Life Science UV/VIS Spectrophotometer (Beckman Coulter GmbH, Germany). We calculated the ratio of 260 nm/280 nm and 260 nm/230 nm to obtain information about the RNA purity. The RNA concentration ranged from 608 ng/µl to 2,689 ng/µl, and we performed agarose gel electrophoresis to visualize ribosomal bands and to assess the quality of the isolated RNA.

## cDNA synthesis

We initiated reverse transcription with the miScript II RT Kit *(Qiagen, Hilden, Germany)*. We prepared a master mix consisting of 5 × miScript HiSpec buffer, 10 × miScript Nucleics mix, miScript Reverse Transcriptase mix, Rnase-free water and template RNA. Typically, we used 1 µg of total RNA to initiate the reaction. In the case of blood/plasma samples the concentration of RNA was very low and typically, we could not quantify its concentration reliable. Therefore, we used 1.5 µl of the eluate from the spin column for the isolation of RNA (see above) for RT. We performed the RT at 37 °C for 60 min followed by a cycle at 95 °C for 5 min in the C1000 Touch Thermal cycler *(Biorad, California, USA).*

## Quantitative PCR of 15 PNF associated miRNAs

Tissue RNA extracts: We performed qPCR with the miScript SYBR Green PCR Kit *(Qiagen, Hilden, Germany)*. The reaction mix consisted of 12.5 µl 2 × QuantiTect SYBR Green PCR Master Mix, 2.5 µl 10 × miScript SYBR Universal Primer, 6.5 µl Rnase-free water, 2.5 µl 10 × miScript Primer Assay and 1 µl tissue derived template cDNA (~ 3 ng). The total reaction volume is 25 µl.

Blood samples: We added 200 µl of water to the entire cDNA prepared from individual plasma samples (approximately 20 µl) and used 4 µl of cDNA template and 3.5 µl water to the PCR-Mix to initiate the reaction as detailed above.

We performed the PCR on a C1000 Touch Thermal cycler and a CFX96 Real-Time system *(Biorad, California, USA)*. We used the Bio-Rad CFX Manager 3.1 software *(Biorad)* to analyse the data and to visualize the amplification curves. Given in supplementary Table S2 and S3 are the conditions of the PCR reactions and the primer sequences.

**Table 1:** PNF regulated miRNAs in ALF, severe drug induced liver injury and fatty liver disease

PNF regulated miRNAs of the present study	Independent confirmation of PNF regulated miRNAs in various pathological conditions	Serum / Tissue	PubMed
miRNA 122-5p miRNA 122-3p	acute liver failure and spontaneous remission from ALF	S↑, T↑	[50]
	severe drug induced liver injury (DILI) progressing to ALF	S↑	[56]
	Steatosis, NASH	S↑, T↓	[61, 72]
miRNA 125b-5p	HBV-ACLF	T↓	[51, 73]
	ALF, Regulator of cell death	T↓	[74]
	sDILI / ALF	S↑	[56]
	NASH	S↑	[61]
miRNA 192-5p	sDILI / ALF	S↑	[56]
	MASLD, MASH	S↑	[61, 72]
	Apoptosis	T↓	[75]
miRNA 27b-3p	sDILI / ALF	S↑	[56]
	Steatosis	S↑	[72]
miRNA 103a-3p	sDILI / ALF	S↑	[56]
	Steatosis	T↑	[76]
miRNA 194-5p	sDILI / ALF	S↑	[56]

We compared PNF associated miRNAs to published findings for acute liver failure (ALF) and severe drug induced liver injury (DILI) cases. Note, the DILI cases are mainly due to acetaminophen overdose. *S* Serum, *T* Tissue, *P* Plasma *↑* upregulation, *↓* downregulation

**Data-analysis:** We applied the 2^−(∆∆CT)^ method to calculate changes of disease regulated miRNAs using the following formula:

- ∆CT = CT (miRNA of interest) – CT (reference gene)
- ∆∆CT = ∆CT (patient sample) – ∆CT (healthy control)
- Fold change = 2^.^−(∆∆CT)^

We used RNU6B as a reference gene to determine the ∆Ct value of a miRNA of interest of either fresh or FFPE liver tissue material. In the case of plasma extracts the mean of miRNA16-5p and Cel-miR-39-3p served as a reference gene. To evaluate the regulation of miRNAs among PNF cases and to determine fold changes, we calculated the ∆CT values of N = 11 individual controls and used the average ∆CT for comparisons with individual PNF cases. Additionally, we investigated the regulation of miRNAs in the circulation pre- and post-surgery (up to 3 days) and compared the results with tissue extracts of the same patients.

We applied the Shapiro–Wilk method to test for normality. Depending on the data distribution, we used the paired/unpaired t-test or the non-parametric Mann–Whitney-Test or Wilcoxon matched-pairs signed rank test. A * denotes a significant *p*-value < 0.05, **p < 0.01, ***p < 0.001 and ****p < 0.0001. We performed all statistical computations with the GraphPad Prism software version 8.4.3.

## Search for miRNA gene targets

Based on a fatty allograft PNF disease model [14] we performed a genome wide scan to identify target genes of significantly regulated miRNAs in PNF. Among the highly regulated miRNAs, we focused on those with an established role in hepatic lipid metabolism, liver injury and regeneration as well as programmed cell death. Based on genome wide miRNA scans, we selected 15 miRNAs that were highly regulated and performed gene ontology annotations to convert rat miRNAs into their human orthologues using the g-profiler program [26]. Subsequently, we queried the miRNet public repository to identify potential genes targeted by the selected miRNAs [27]. We compared the list of potential targets with significantly regulated genes which we identified in fatty allograft failing livers of the rat (PNF study) and searched for common targets. This defined 2,307 genes, and we visualized the miRNA-target gene networks with the Cytoscape software (U.S. National Institute of General Medical Sciences, NIGMS). Additionally, we evaluated the biological functions of the regulated target genes with the gene ontology tool Metascape [28] and David database [29] and created visual networks.

## Rat serum

Details regarding the animal study are given in our recent publication (see [14]), and the study is reported in accordance with ARRIVE guidelines. Ethical approval was granted by the animal welfare ethics committee of the State of Lower Saxony, Germany (“Lower Saxony State office for Consumer Production and Food Safety” [LAVES]). The approval ID is Az: 33.14–42,502-04–13/1258. All methods were carried out in accordance with relevant guidelines and regulations.

Blood samples were obtained from recipients of healthy donor allografts (CTx) and fatty liver allografts (MTx) post-OLT on day 1, 3, 7 and 14. Serum was prepared from whole blood using standard procedures as described in Kulik et al. 2024 [14]. We performed a genome wide search for regulated miRNA. This defined differentially expressed miRNAs in the circulation of fatty allograft recipient animals, and we selected 15 miRNAs for their time dependent regulation and clinical validation.

## Results

**Fig. 1:**
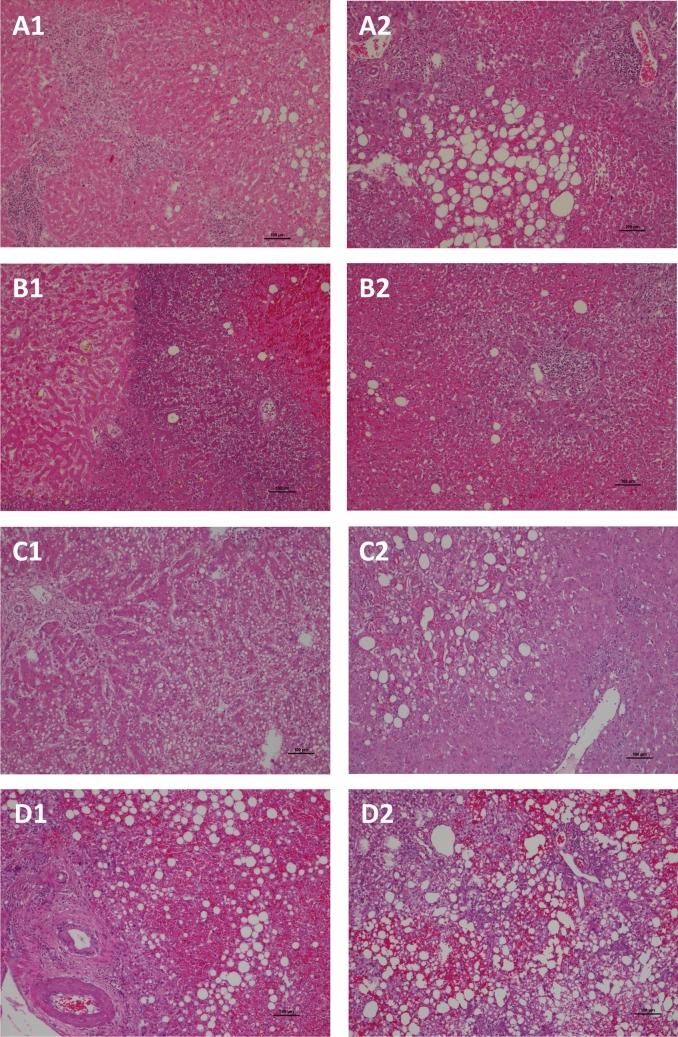
Histopathology of fatty allograft associated PNF cases. Depicted are 7 individual H&E-stained liver sections of PNF cases (panel B1 and B2 stem from the same individual) with various grades of hepatic steatosis, inflammation, necrosis and fatty liver dystrophy. A description of the individual cases is given in the result section

Based on findings from a disease model of fatty allograft associated PNF [14], we selected 15 highly regulated miRNAs with known functions in the control of lipid metabolism, apoptosis, acute liver failure and liver regeneration (Table 1). Shown in Fig. 1 are H&E-stained liver sections of PNF allografts with varying degree of hepatic steatosis, inflammation and necrosis. Case A1 is a 43 year (y) old male who received a fatty allograft. Histology of the first allograft evidenced marked centrilobular and subcapsular map-like necrosis excessive inflammatory infiltrates, shrunken and vacuolated hepatocytes and macrovesicular steatosis (Case A1). Case A2 is a 58y old female. Note the marked macrovesicular steatosis, the centrilobular necrosis, the partial destruction of portal fields and extra-hepatic bile duct necrosis. Panel B1 and B2 refer to a 39y old female. Here, histology revealed subtotal necrosis indicative for excessive reperfusion injury. A further example relates to a 40y old female, and the liver section in C1 shows mixed micro- and macrovesicular steatosis, parenchymal necrosis and ischemia/reperfusion injury. Panel C2 refers to a 57y old male patient, and this liver section shows primarily macrovesicular steatosis, perivenular cholestasis with occasional lymphocytic infiltrates. Depicted in Fig. 1 panel D1 is the case of a 60y old male with marked portal inflammatory infiltrates, extensive lobular necrosis and macrovesicular steatosis of the fatty allograft. Case D2 refers to a 49y old female, and the allograft shows excessive macrovesicular steatosis, fresh hemorrhage, centrilobular necrosis and acute fatty liver dystrophy.

## PNF-associated miRNAs

To validate PNF associated miRNAs, we analyzed fifty-nine FFPE tissue blocks of 29 PNF cases and compared the data to 11 individual controls, i.e. morphological normal liver resection material obtained in the course of an elective hepatobiliary surgery (supplementary Table S1). Furthermore, we addressed the question whether the degree of hepatic steatosis influenced their regulation.

**Fig. 2:**
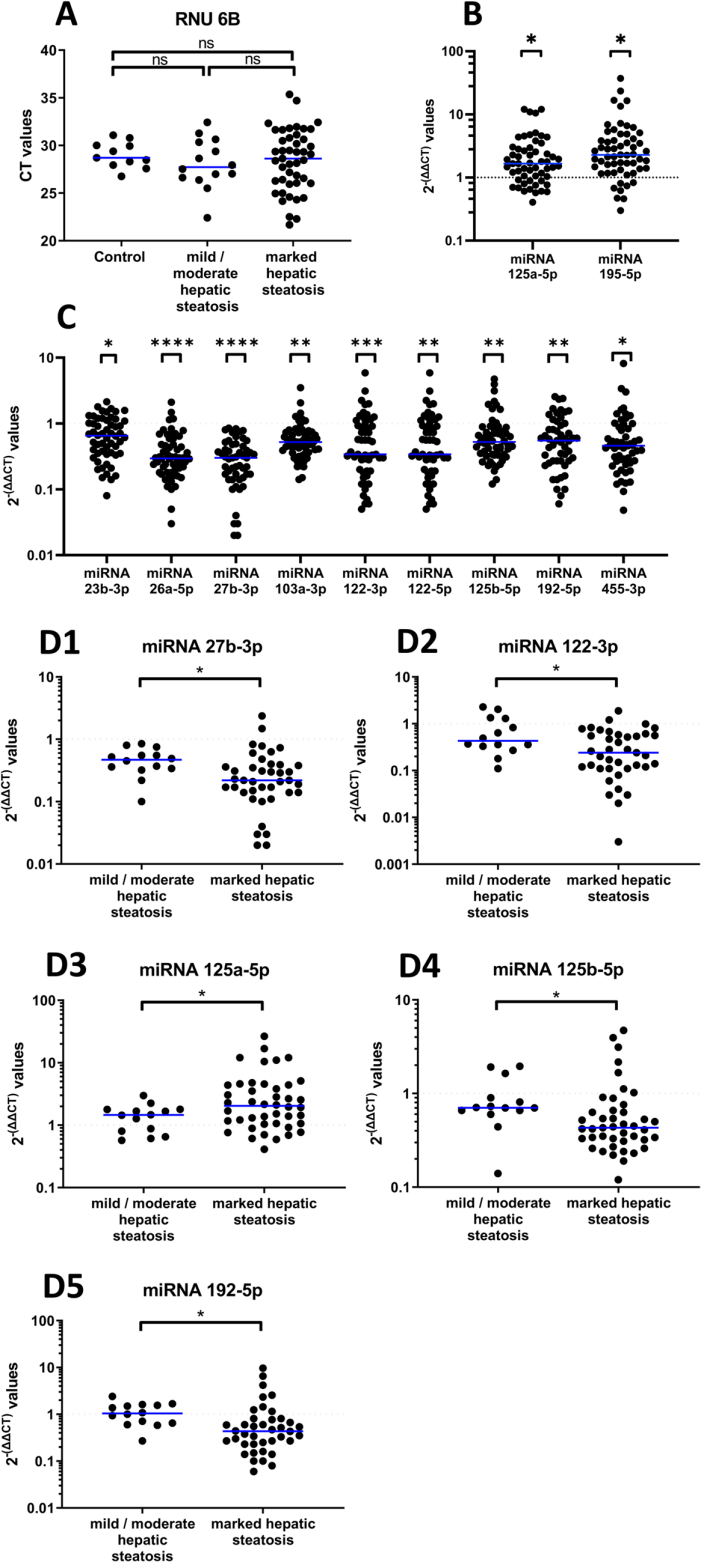
Regulation of miRNAs in liver tissue of fatty allograft associated PNF cases. MiRNAs were extracted from FFPE sections. The data are 2^−(∆∆CT)^-values for significantly regulated miRNAs. We used Shapiro–Wilk normality test and unpaired t-test or Mann–Whitney-Test. Shown are median and p-values. **A** The expression of the RNU6B housekeeping gene is similar in control and PNF cases. **B** miRNA-125a-5p and miRNA-195-5p are significantly upregulated in liver tissue of fatty allograft associated PNF cases. **C** miRNA-23b-3p, miRNA-26a-5p, miRNA-27b-3p, miRNA-103a-3p, miRNA-122-3p, miRNA-122-5p, miRNA-125b-5p, miRNA-192-5p and miRNA-455-3p are significantly repressed in PNF cases. **D** Hepatic steatosis grade dependent regulation of miRNA-27b-3p, miRNA-122-3p, miRNA-125a-5p, miRNA-125b-5p and miRNA-192-5p among PNF cases. Note, significance is calculated from ∆CT-values (p = 0.03, p = 0.02, p = 0.04**,** p = 0.01, p = 0,02)

First, we considered the expression of the house keeping gene RNU 6B. Its expression did not differ between controls and PNF cases irrespective of the degree of hepatic steatosis (Fig. 2A). Therefore, the selection of the housekeeping gene was justified and used as a “normalizer” in qPCR assays.

Second, we considered the interpatient variability in the expression of individual miRNAs and found all to behave similar (supplementary Table S4). Therefore, we excluded sampling bias as a possible confounder.

Third, we computed the 2^−(∆∆CT)^-values of miRNAs among PNF cases and found 11 out of 15 miRNAs to be significantly regulated (Fig. 2B&C, non-regulated miRNAs in supplementary Figure S2A). Except for miRNA-125a-5p and miRNA-195-5p (Fig. 2B) the miRNAs were repressed in expression when compared to morphologically normal tissue as exemplified for miRNA-26a-5p and miRNA-27b-3p which were repressed to about 30% of controls (p < 0.0001, Fig. 2C). Note, the latter two miRNAs were highly repressed in 80% and 85% of cases.

Fourth, we addressed the question whether the degree of hepatic steatosis influenced expression of PNF-associated miRNAs. While for the majority of PNF regulated miRNAs the expression remained alike (supplementary Figure S2B), we found miRNA-27b-3p, miRNA-122-3p, miRNA-125a-5p, miRNA-125b-5p and miRNA-192-5p to be significantly influenced by the degree of hepatic steatosis (Fig. 2D). The results suggest that hepatic steatosis aggravated the repression of these miRNAs.

Fifth, we compared the regulation of PNF associated miRNAs in clinical samples to findings obtained from the animal study, and the results were comparable (Table 2). For instance, in rat liver and human PNF cases miRNA-122-5p was repressed to 35% and 34% of controls. Notwithstanding, there are also significant differences in PNF associated miRNA regulations between human cases and the animal model. Specifically, let-7b-5p, miRNA-125a-5p, miRNA-126-3p, miRNA-194-5p and miRNA-195-5p were oppositely regulated between clinical cases, and the animal model and the changes were more pronounced in the animal PNF-model.

**Table 2:** Regulation of PNF associated miRNAs in human and rat liver tissue

	PNF rat Mean, 95% CI	PNF human Mean, 95% CI	PNF human p-value
*Let-7b-5p*	15% (13–18%)	211% (100–302%)	0.19
*miRNA-19b-3p*	11% (10–12%)	51% (41–70%)	0.16
*miRNA-23b-3p*	4% (4–5%)	65% (48–89%)	0.02
*miRNA-26a-5p*	6% (6–7%)	30% (24–36%)	< 0.0001
*miRNA-27b-3p*	48% (25–100%)	30% (21–36%)	< 0.0001
*miRNA-103a-3p*	6% (6–7%)	5% (43–59%)	0.002
*miRNA-122-3p*	4% (4–4%)	28% (17–52%)	0.0004
*miRNA-122-5p*	35% (29–45%)	34% (30–73%)	0.006
*miRNA-125a-5p*	4% (4–4%)	166% (130–215%)	0.04
*miRNA-125b-5p*	3% (3–3%)	53% (42–66%)	0.009
*miRNA-126-3p*	3% (3–3%)	154% (119–195%)	0.27
*miRNA-192-5p*	21% (18; 25)	55% (35–67%)	0.006
*miRNA-194-5p*	7% (6; 8)	105% (77–137%)	0.59
*miRNA-195-5p*	4% (4; 4)	228% (172–301%)	0.02
*miRNA-455-3p*	8% (7; 8)	46% (33–54%)	0.02

The data are mean and 95% CI-values

## Ischemia injury

**Fig. 3:**
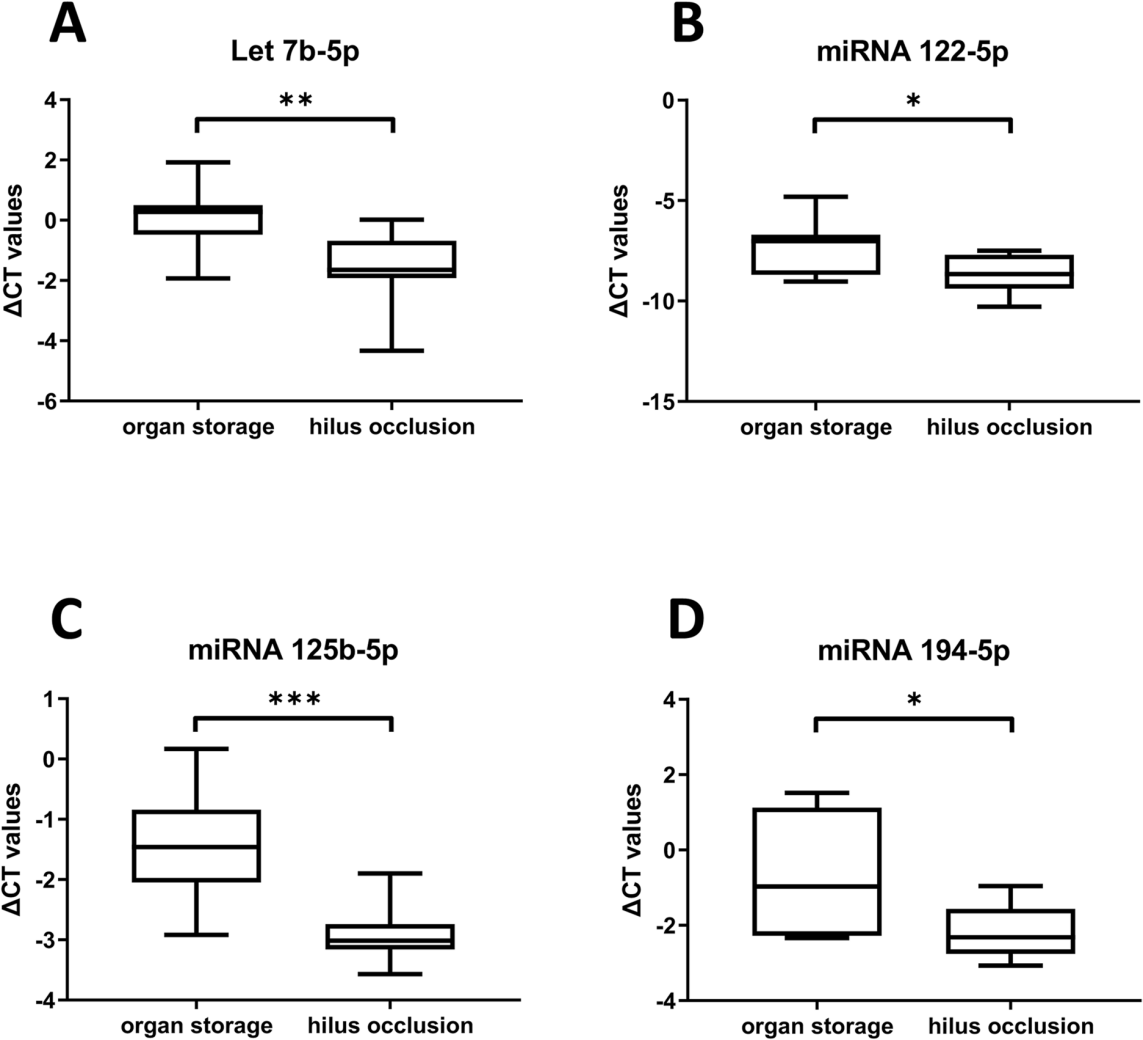
Ischemia injury regulated miRNAs. MiRNAs were extracted from T0 liver biopsies, i.e. prior to OLT and intraoperative following hilus occlusion in the course of hepatectomy. The data are ∆CT-values for significantly regulated miRNAs. Importantly, subtracting the high abundance RNU6B reference gene from low abundance miRNA transcripts yielded negative ∆CT values. We compared expression of a given miRNA in T0 biopsies (organ storage prior to OLT = cold ischemia) to its expression following hilus occlusion (= warm ischemia), and this caused increased expression of let-7b-5p, miRNA-122-5p, miRNA-125b-5p and miRNA-194-5p. Note, a reduction in the ∆CT value implies an increase in transcript expression. We used Shapiro–Wilk normality test and unpaired t-test (p-values: p = 0.01, p = 0.03, p = 0.0007**,** p = 0.02)

**Fig. 4:**
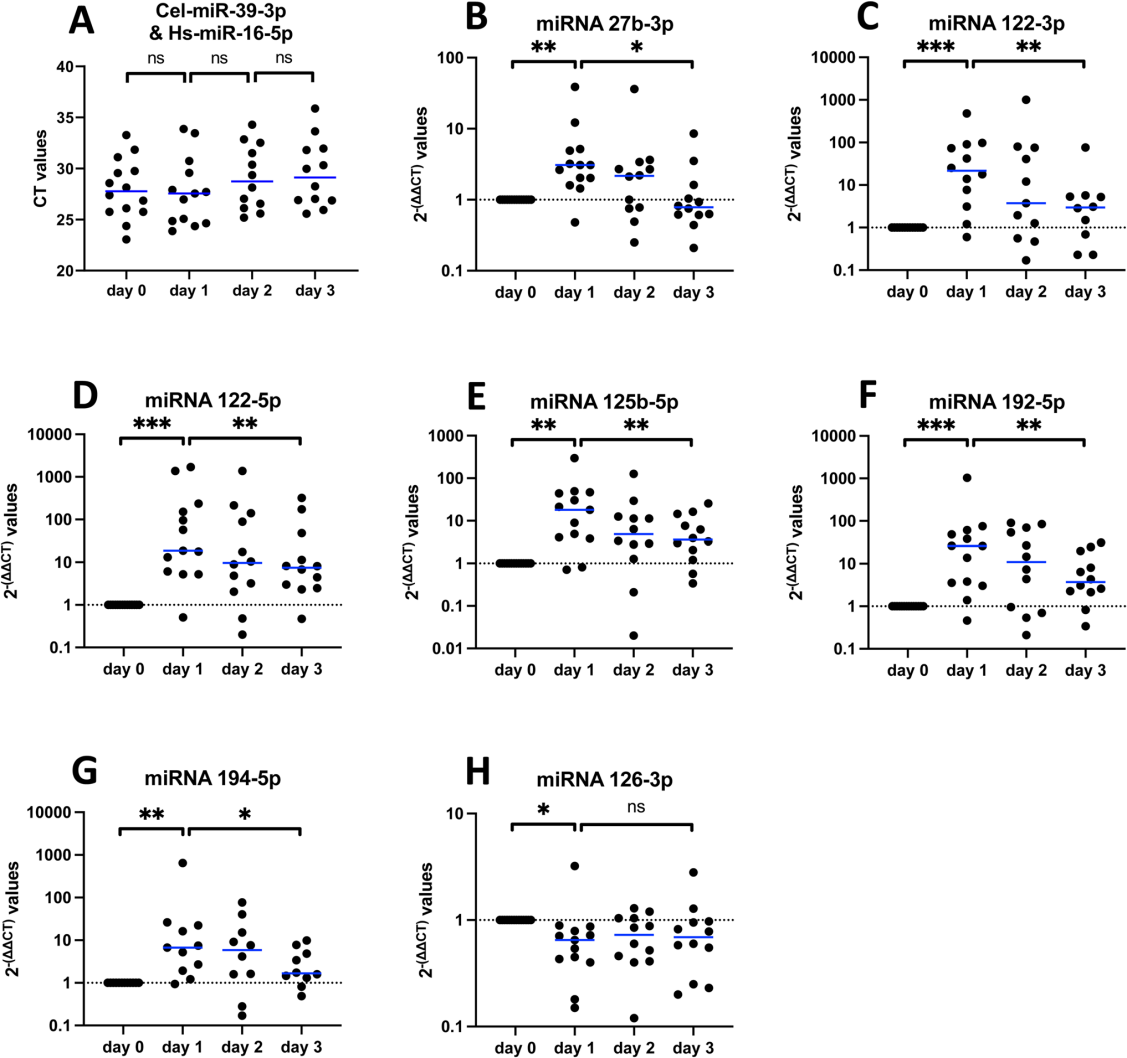
Blood-borne miRNAs after OLT of healthy allografts and tumor liver resections. MiRNAs were extracted from plasma prior to and post-surgery. The data are 2^−(∆∆CT)^-values for significantly regulated miRNAs. Except for miRNA-126-3p all miRNAs are upregulated on day 1 post-surgery and decline thereafter. We used Shapiro–Wilk normality test and paired t-test or Wilcoxon matched-pairs signed rank test. **A** Constant expression of the housekeeping gene miRNA-16-5p and Cel-miRNA-39-3p in pre- and post-surgery. **B–G **Upregulated: miRNA-27b-3p, miRNA-122-3p, miRNA-122-5p, miRNA-125b-5p, miRNA-192-5p and miRNA-194-5p. **H** Downregulated miRNA-126-3p

To assess the effects of ischemia injury on the regulation of PNF associated miRNAs, we evaluated their expression in biopsies of liver allografts (N = 7, supplementary Table S5A) prior to transplantation. Additionally, we obtained intraoperative biopsies after hilus occlusions during hepatic surgery (N = 10, supplementary Table S5B) and compared the regulation of miRNAs of donor liver biopsies during organ storage to intra-operative liver biopsies taken from patients undergoing elective hepatobiliary surgery. The data shown in Fig. 3 are ∆CT values for significantly upregulated miRNAs following hilus occlusion. For instance, miRNA-122-5p was nearly threefold upregulated in liver tissue following ischemia injury (median: 2.6-fold, Fig. 3B). This miRNA is a well-known marker of liver injury. Independent research demonstrated miRNA-122-5p to be highly enriched in the nucleus of liver cells and to block activity of the cell survival oncomiR miR-21 at the posttranscriptional level [30]. Since ischemia injury is associated with marked cellular damage, its release into circulation was expected. Indeed, we found blood borne miRNA-122-5p to be 19-fold upregulated on day 1 post-surgery, and this represents a > 600% increase of this miRNA when compared to its induced tissue expression (see Fig. 4).

Additionally, tissue expression of let-7b-5p, miRNA-125b-5p and miRNA-194-5p was significantly upregulated by about threefold, and these miRNAs are known to augment inflammation and hepatic stellate cell activation. For comparison, the data of non-significantly regulated miRNAs are given in supplementary Figure S3.

## miRNA in the systemic circulation following reperfusion injury of clinical cases

Ischemia/reperfusion (IR) injury is an unavoidable process in hepatic surgery and to determine whether the selected miRNA biomarkers are also regulated in response to IR injury, we investigated their regulation in blood samples taken prior to (T0) and post-surgery on day 1, 2 and 3.

We evaluated 14 patients of which one-half were OLT cases, and the other half consisted of elective surgeries for primary or secondary liver malignancies (supplementary Table S6). We obtained serial blood samples from the same patients and determined CT-values by the 2^−(∆∆−CT)^-method. We used the average CT-values of miRNA-16-5p and Cel-miRNA-39-3p as reference genes (Fig. 4A), and independent research demonstrated the advantageous of applying the average of two reference genes for data analysis [31–33]. The time course of individual blood borne miRNAs is shown in Fig. 4B–G, and 6 out of 15 PNF associated miRNAs, i.e. 27b-3p, 122-3p, 122-5p, 125b-5p, 192-5p and miRNA-194-5p were significantly upregulated in plasma by a range of 7- 26-fold post-surgery. Importantly, we observed opposite regulation of tissue (Fig. 2) and blood borne miRNAs, and this highlights their sensitivity to IR injury. All regulated miRNAs returned to pre-surgery or even below T0 expression values on day 3 post-surgery, and unchanged miRNAs are given in supplementary Figure S4. However, miRNA-126-3p remained consistently repressed (Fig. 4H) and experimental research demonstrated this miRNA to be a target Hoxb6. This transcription factor controls expression of SOX9 in liver progenitor cells which are destined to replace damage cells following CCL4 liver injury of mice [34]. Therefore, a regulatory loop exists between miRNA-126-3p, Hoxb6 and SOX9, and it is tempting to speculate that repressed miRNA-126-3p serum levels in clinical samples signify delayed liver regeneration.

Additionally, we searched for blood borne miRNAs either regulated in OLT or tumor associated surgery. Depicted in supplementary Figure S5, panel A are miRNAs which were explicitly regulated in tumor liver resection cases, i.e. miRNA-19b-3p, miRNA-125a-5p and miRNA-126-3p, and these function in wound repair and fibrosis, liver regeneration and metabolic disease. For instance, overexpression of miRNA-125a-5p supports liver regeneration [35]. Conversely, miRNA-27b-3p and miRNA-194-5p were specifically regulated in OLT (supplementary Figure S5, panel B), and these function in inflammation and rejection of the graft [36].

To evaluate different grades of hepatic steatosis on the regulation of blood borne miRNA, we compared plasma samples of patients diagnosed with mild to moderate steatosis (N = 7) to cases of marked steatosis (N = 7). Obviously, the number of patients is small, but even so, miRNA-103a-3p reached statistical significance, and this miRNA promotes hepatic steatosis by repressing the expression of palmitoyl-CoA oxidase [37]. Furthermore, the expression of miRNA-26a-5p, miRNA-27b-3p, miRNA-103a-3p and miRNA-122-5p tended to be higher in cases of marked steatosis (supplementary Figure S6), however, did not reach statistical significance.

## Serum miRNAs in a rat fatty allograft OLT model

**Fig. 5:**
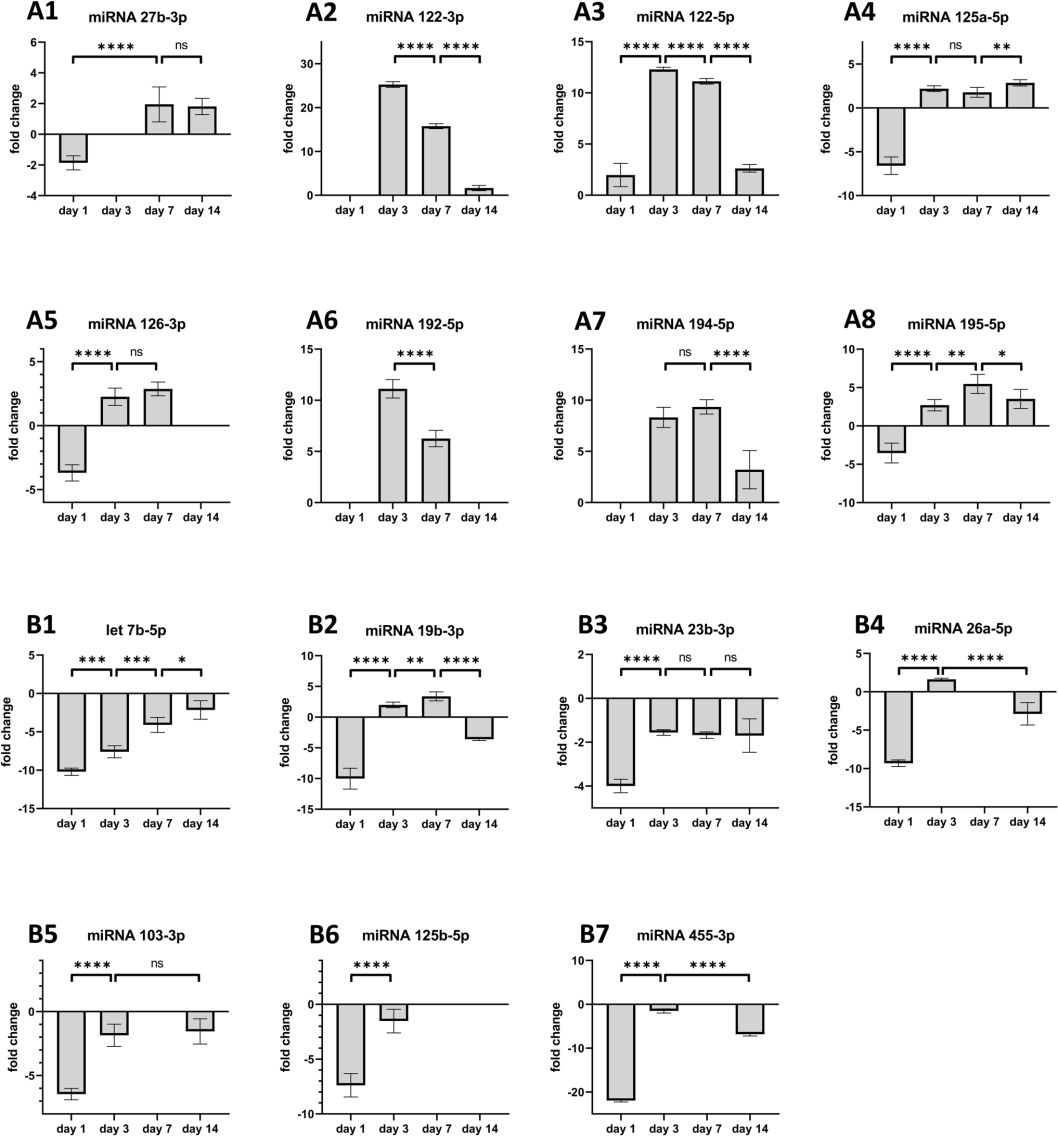
miRNAs in the systemic circulation after OLT of healthy allografts in a rat liver transplantation model. MiRNAs were extracted from rat serum post-surgery on days 1, 3, 7 and 14. The data are fold changes relative to non-transplanted controls. Depicted in panel A and B, respectively are predominantly up- and down-regulated miRNAs following liver transplantation of healthy allografts

We recently reported the development of a PNF fatty allograft disease model [14] and determined the regulation of 15 miRNAs in blood samples of rats following liver transplantation on days 1, 3, 7 and 14 post-surgery (Fig. 5). We compared their expression in serum of non-transplanted Chow-fed controls to OLT of healthy allografts. The data are shown as fold changes, and we observed upregulation of miRNA-27b-3p, miRNA-122-3p, miRNA-122-5p, miRNA-125a-5p, miRNA-126-3p, miRNA-192-5p, miRNA-194-5p and miRNA-195-5p in the circulation (Fig. 5A, range 25 to twofold). Conversely, miRNA-7b-5p, miRNA-19b-3p, miRNA-23b-3p, miRNA-26a-5p, miRNA-125b-5p and miRNA-455-3p were downregulated (Fig. 5B, range 22 to 1.5-fold). Although the latter miRNAs were below the expression of Chow-fed controls, it is obvious that their expression increased with time, thus implying improved liver function following OLT.

**Fig. 6:**
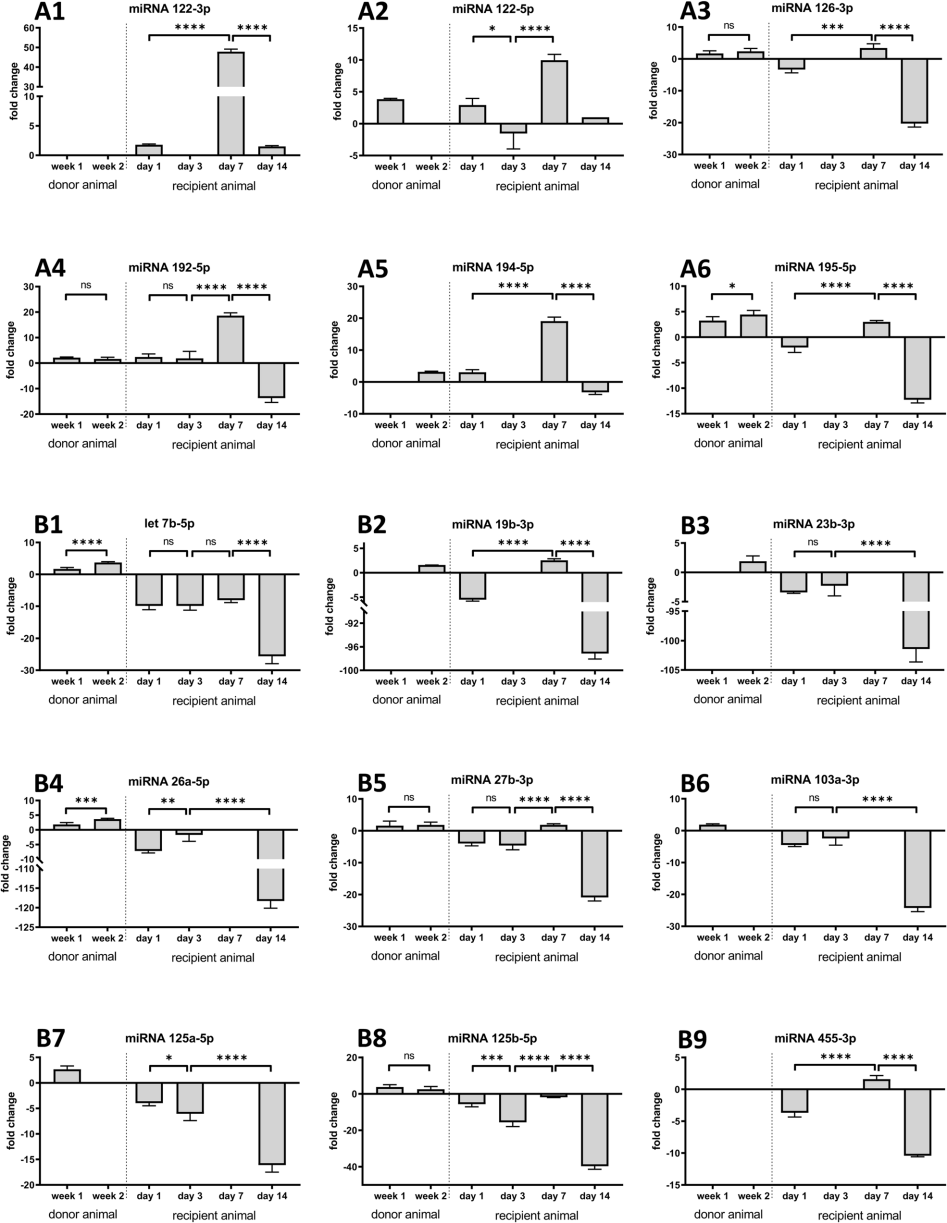
Regulation of serum miRNAs after OLT of fatty allografts. MiRNAs were extracted from rat serum samples of donor animals on day 7 and 14 (left panel). Furthermore, we show their regulation in recipient animals, post-surgery on days 1, 3, 7 and 14. The data are fold changes by comparing their expression in serum samples of recipients of healthy donor and fatty allografts. Depicted in panel A and B, respectively are predominantly up- and down-regulated miRNAs following liver transplantation of fatty allografts

Additionally, we compared the regulation of PNF associated serum miRNAs in donor animals on a CHOW and MCD diet for 7 and 14 days (Fig. 6, left panels). Essentially, we observed mild increases of these miRNAs (range 2 to fourfold) in MCD fed animals. This demonstrates their fatty liver associated regulation.

Subsequently, we compared the regulation of PNF associated miRNAs in serum of rats following OLT of healthy and fatty allografts. Shown in the right panels of Fig. 6 are serum miRNAs regulated in recipient animals following OLT of fatty allografts. The data are fold changes by comparing them to healthy allografts, and we divided the results into up-regulated (Fig. 6A) and downregulated (Fig. 6B) miRNAs post-OLT for up to 7 days. We observed marked induction of miRNA-122-3p and miRNA-122-5p, i.e. 48- and tenfold induced, respectively in fatty allografts on day 7 post-OLT. Similarly, we observed a 19-fold increased expression of miRNA-194-5p in fatty allografts on day 7 post-OLT. Furthermore, miRNA-126-3p, miRNA-192-5p and miRNA-194-5p were upregulated on day 1, 3 and 7 but downregulated on day 14 post-OLT.

Conversely, Let-7b-5p, miRNA-27b-3p, miRNA-103-3p, miRNA-125a-5p, miRNA-125b-5p were downregulated in recipient animals of fatty allografts. Meanwhile, miRNA-19b-3p, miRNA-23b-3p and miRNA-26a-5p were unchanged up to 7 days post-OLT but declined thereafter (Fig. 6B).

Except for miRNA-122, all serum miRNAs were significantly downregulated on day 14 post-OLT (Fig. 6). Thus, fatty allograft OLTs are hallmarked by repression of PNF associated miRNAs, and the results are similar to clinical samples (Fig. 2) on day 14 post-OLT.

## miRNA gene-target networks in PNF

**Fig. 7:**
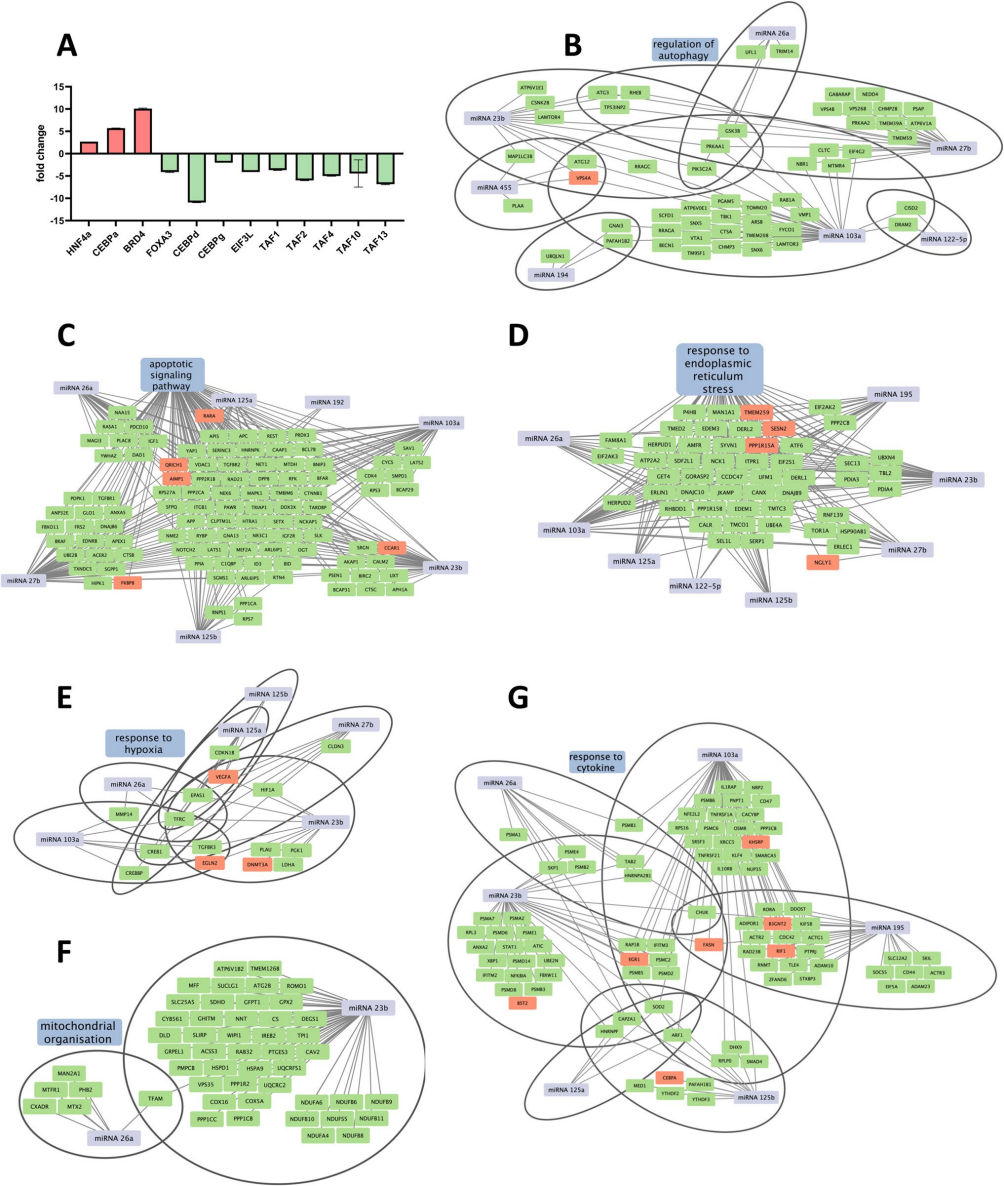
Construction of miRNA-gene regulatory networks in rat fatty liver associated PNF cases. We performed genomics in healthy allografts and fatty allografts associated PNF cases. Shown are miRNA-gene networks in failing livers following fatty allograft transplantation. **A** Regulation of transcription factors and transcriptional repressors. **B** Regulation of miRNA-gene networks. Shown are Cytoscape visualized networks of autophagy. **C** Apoptotic signaling pathway. **D** Response to endoplasmic reticulum stress. **E** Response to hypoxia. **F** Mitochondrial organization. **G** Response to cytokines

We performed a genome wide scan to identify genes regulated in PNF cases [14], and for this purpose compared the transcriptomes of rat liver following OLT of healthy donor allografts (CTx) to PNF cases. This revealed 2450 differentially expressed genes (DEGs) of which 2,215 or nearly 91% were repressed. Therefore, PNF caused an unprecedented repression of the transcriptome and involved various components of the general transcription machinery including the CAAT enhancer binding proteins, TATA-Box binding protein associated factors, i.e. TAF-proteins, and various liver enriched transcription factors (HNF4alpha and FOXA3, Fig. 7A) [38, 39]. Strikingly, the BRD4 bromodomain and extra-terminal domain transcriptional activator is highly induced (> tenfold) and was recently shown to be a key player in the global loss of activity of the transcriptional machinery in damaged livers [40]. Typically, BRD4 binds to acetylated lysine residues of the chromatin (super-enhancers) and supports transcriptional activation of genes.

Furthermore, we queried the miRnet public data base to search for experimentally proven targets of 15 PNF associated miRNAs and compared the results to DEGs identified in PNF livers. We focused on target genes coding for hepatic lipid metabolism, liver injury and regeneration as well as programmed cell death and report results for 363 target genes. Depicted in Fig. 7 are the networks for autophagy (panel 7B), apoptotic signaling (panel 7C), response to endoplasmic reticulum stress (panel 7D), response to hypoxia (panel 7E), mitochondrial organization (panel 7F) and response to cytokines (panel 7G). Note, the majority of target genes are repressed, and 4/5 of the regulated genes are experimentally proven targets while the remaining are predicted targets.

## Discussion

Based on findings from a rat liver transplant model [14], we aimed at validating fatty allografts associated miRNAs predictive for PNF. We confirmed clinical significance for 11 miRNAs, of which 9 and 2, respectively were down- and upregulated (Table 2 and Fig. 2). Our study revealed the significant relationship between the degree of hepatic steatosis and the repression of miRNA-27b-3p, miRNA-122-3p, miRNA-125b-5p and miRNA-192-5p in liver tissue of clinical PNF-cases (Fig. 2D). Note, under hypoxic conditions and through upregulation of the transcription factor PPARγ, miRNA-27b plays an essential role in lipid metabolism [41, 42] while silencing of miRNA-125b-5p promotes liver fibrosis in MASLD via integrin α8-mediated activation of the RhoA signaling pathway [43]. Furthermore, repressed miRNA-192-5p aggravates lipid deposition by controling the expression of stearoyl-CoA desaturase 1 [44, 45].

Additionally, we explored the regulation of PNF associated miRNAs in liver biopsies taken prior to OLT and intraoperatively following hilus occlusion. We found 4 miRNAs (let-7b-5p, miRNA-122-5p, miRNA-125b-5p and miRNA-194-5p) significantly upregulated when biopsies following hilus occlusion were compared to T0 liver biopsies (Fig. 3). Moreover, we identified 6 up- and 1 downregulated miRNA in post-surgery blood samples of successfully performed OLTs and tumor liver resection cases (Fig. 4). Importantly, these miRNAs were oppositely regulated when compared to PNF cases (Fig. 2). Therefore, we demonstrate selectivity and specificity and clinical relevance for the majority of the miRNAs. Table 3 summarizes the 15 miRNAs and their regulation in FFPE-PNF-tissue (Fig. 2), pre- and intraoperative liver biopsies (Fig. 3) and blood samples taken from patients which underwent elective hepatobiliary surgery (Fig. 4).

**Table 3:** Regulation of miRNAs in human fatty allograft associated PNF cases, in T0 liver biopsies of healthy allografts prior to OLT and hepatectomies of tumor resection

miRNA	Tissue expression in fatty allograft associated PNF	OLT healthy allografts and hepatectomy of neoplasms	
		Plasma	Liver tissue
miRNA 122-5p	↓**	↑***	↑*
miRNA 125b-5p	↓**	↑**	↑***
miRNA 27b-3p	↓****	↑**	ns
miRNA 122-3p	↓***	↑***	ns
miRNA 192-5p	↓**	↑***	ns
miRNA 26a-5p	↓****	ns	ns
miRNA 23b-3p	↓*	ns	ns
miRNA 103a-3p	↓**	ns	ns
miRNA 455-3p	↓*	ns	ns
miRNA 125a-5p	↑*	ns	ns
miRNA 195-5p	↑*	ns	ns
miRNA 194-5p	ns	↑**	↑*
miRNA 126-3p	ns	↓*	ns
Let 7b-3p	ns	ns	↑**
miRNA 19b-3p	ns	ns	ns

In Table 1, we summarize the various functions of PNF associated miRNAs in the control of lipid metabolism, acute liver failure, IR injury and liver regeneration. The regulation of miRNA-122 and its two mature products, i.e. miRNA-122-3p and -5p is an interesting example [46]. Although abundantly expressed in the liver, miRNA-122-3p is not significantly regulated in liver tissue resection material (Fig. 3); however, is highly upregulated in blood samples following surgery (Fig. 4). On the first day post-surgery, its regulation ranged between 0.7 and 270-fold across individual patients, and this miRNA serves as a marker of liver cell damage. In contrast, miRNA-122-5p is mildly but significantly upregulated in intra-operative biopsy samples following hilus occlusion (median = 2.6-fold) and markedly increased in blood samples of the same patients (median = 20-fold). miRNA-122 is essential for liver metabolic homeostasis and lipid metabolism. It exerts anti-inflammatory and anti-fibrotic properties and blocks viral replication in hepatocytes [47, 48]. Notwithstanding, one report suggests liver injury-induced release of miRNA-122 to stimulate pulmonary inflammation [49]. Typically, its expression is low in serum but highly upregulated during liver injury. Interestingly, in patients with spontaneous recovery from acute liver failure, miRNA-122 is significantly upregulated in serum and liver tissue when compared to no recovered patients. This implies an important role of this miRNA in instructing liver regeneration [50].

A further example relates to miRNA-125b-5p which was reported to alleviate acute liver failure by regulating the Keap1/Nrf2/HO-1 pathway [51]. Furthermore, this miRNA protects from reperfusion injury by inhibiting TRAF6 and NF-κB signaling [52].

Unlike preclinical PNF cases, let-7b-5p is regulated in human liver tissue, but not in blood samples, and this miRNA inhibits cell proliferation [53]. Notwithstanding, one study identified repressed let-7b blood levels in children diagnosed with progressive familial intrahepatic cholestasis [54]. In vitro, this miRNA inhibits hepatic stellate cell activation and therefore plays a role in fibrosis [55].

A recent review summarized significantly regulated miRNAs in human acute liver failure (ALF) cases [56]. The review is based on 21 independent studies and primarily describes findings for acetaminophen overdose and drug induced liver injury (DILI) cases as well as viral liver diseases. Of the ALF serum and plasma regulated miRNAs, 53% are common to our study, i.e. 8/15 miRNA, and this demonstrates relevance of these miRNAs in acute liver failure across independent clinical studies. Although the causes of ALF and PNF are different in nature, i.e. drug induced versus fatty allograft associated PNFs, the results underscore the clinical relevance of the selected miRNAs and their utility as commonly regulated biomarkers in PNF and ALF.

Table 1 compiles miRNAs commonly regulated between clinical ALF and fatty allograft associated PNF cases and highlights their basic function in liver biology. For instance, miRNA-27b-3p regulates mitochondrial biogenesis [57] and targets several key lipid-metabolism genes [58]. This miRNA is highly repressed in fatty allograft associated PNF cases (Fig. 2) and given its role in mitochondrial biogenesis, its repression might be regarded as an adaptive response. Indeed, an inverse relationship exists between miRNA-27b expression and mitochondria content [57]. Similarly, de novo lipogenesis can be inhibited by miRNA-27a. This miRNA alleviates obesity-initiated MASLD by repressing the expression of fatty acid synthase and stearoyl-CoA desaturase [59]. Furthermore, miRNA-27b-5p inhibits PPARγ driven lipogenesis [60].

A further example relates to an identification of circulating miRNAs in MASLD patients. Specifically, Pirola and co-workers investigated serum microRNAs among liver biopsy proven MASLD cases and healthy controls [61]. Of the 84 investigated miRNAs, blood borne miRNA-122, miRNA-192, miRNA-19a and miRNA-19b, miRNA-125b proved to be of diagnostic value. In the present study miRNA-122-5p, miRNA-192-5p, miRNA-125b-5p were highly significantly repressed among fatty allograft associated PNF cases, and their regulation was influenced by the hepatic lipid content (Fig. 2D). Unlike liver biopsy and serum findings for MASLD patients [61] miRNA-19b-3p was not significantly regulated in fatty allograft associated PNF cases (supplementary Figure S2, panel A).

The role of miRNA-192-5p in human diseases is the subject of a recent review, and there is evidence for this miRNA to effect energy metabolism [45]. Downregulation of miRNA 192 causes hepatic steatosis through upregulation of sterol regulatory element binding transcription factor 1 [62]. In the present study, miRNA-192-5p was markedly repressed among fatty allograft associated PNF cases. Its regulation was influenced by the hepatic lipid content (Fig. 2D5) and correlated with the degree of steatosis. Conversely, miRNA-192-5p is significantly upregulated in blood samples of patients following OLT of healthy allografts and patients undergoing hepatectomy (Fig. 4). Moreover, the importance of the HNF4α-miRNA-194/192 signaling axis in maintaining hepatic cell function was demonstrated in liver-specific Hnf4a-null (Hnf4aΔH) mice [63], and miRNA-192-5p and miRNA-194-5p are localized in a cluster. Note, both miRNAs were significantly upregulated in plasma samples following hepatic surgery (Fig. 4), and this demonstrates their diagnostic relevance for distinguishing PNF from liver regeneration cases.

Another miRNA linked to liver regeneration is miRNA-26a. This miRNA is significantly repressed in PNF cases but abundantly expressed in OLT biopsy and blood samples of patients undergoing elective liver surgery (Fig. 3, 4). Independent research demonstrated that the growth factor termed augmenter of liver regeneration (ALR) induces expression of miRNA 26a and stimulated cell proliferation via the microRNA-26a/Akt/cyclin D1 signaling pathway [64]. Conversely, miRNA-26 influences the cross-talk between mdm2 and p53, and its repression stimulates mdm2 expression which inhibits p53 activity [65]. Another study demonstrated downregulation of microRNA-26a to promote mouse hepatocyte proliferation during liver regeneration [66]. Therefore, repressed miRNA-26 supports liver regeneration and can be regarded as an adaptive response to impair programmed cell death.

Liver regeneration is supported by the upregulation of miRNA-125a-5p, and its overexpression in the human liver cell line HL-7702 increased cell viability significantly [35]. In the present study, miRNA-125a-5p was one of the two significantly increased miRNAs (Fig. 2), and we consider its upregulation in fatty allograft associated PNF cases as an attempt to stimulate liver regeneration. Notwithstanding, miRNA-195-5p was also significantly upregulated, and this miRNA promotes hepatic stellate cell activation and liver fibrosis by suppressing PTEN expression in a mouse model of liver damage [67]. Furthermore, downregulation of miR-23b stimulated TGF-β1/Smad3 signaling during the termination stage of liver regeneration [68] and therefore contributes to impaired liver regeneration. Consistent with its function miR-23b is repressed in fatty allograft associated PNF cases (Fig. 2).

Lastly, we observed repressed plasma miRNA-126-3p in post-surgery blood samples of OLTs and tumor liver resection cases (Fig. 4). This miRNA suppresses inflammation in endothelial cells [69], is significantly repressed in higher grade MASLD patients [70], and its repression impairs liver regeneration in mice following partial hepatectomy [71].

Based on their specific regulation by the grade of hepatic steatosis, we propose miRNA-27b-3p, miRNA-122-3p, miRNA-125a-5p, miRNA-125b-5p and miRNA-192-5p as a panel of diagnostic miRNAs to predict fatty allograft associated PNF. Their validation in prospective clinical trials is warranted. In addition, miRNA-26a-5p is highly regulated in most PNF cases (80%) and therefore is a biomarker candidate worthwhile for in depth validation.

## Conclusions

We report an identification of miRNAs significantly associated with fatty allograft associated PNFs. Our findings warrant clinical validation to demonstrate their prognostic value.

## Study limitations

The following caveats need to be considered. First, PNF is a rare and unpredictable event, and therefore it is difficult to design a prospective study. Thus, our findings are based on archived tissue materials. Second, given the retrospective nature of the study, we were unable to control bias, i.e. the outcome was known prior to study initiation. However, we do not consider this bias to be of critical importance for an identification of PNF biomarkers. Third, we were unable to obtain a sufficient number of intraoperative liver biopsies following OLT of healthy allograft. Fourth, although we included all fatty allograft associated PNF cases among 1,200 OLTs performed at our institution, we report a single center study. Nonetheless, the power analysis showed the number of cases to be sufficient to determine statistical significance. Future studies should be based on randomized clinical trials. Fifth, a comparison of miRNAs regulated in donor livers prior to transplantation with cases of hepatic surgery following hilar occlusion is confounded by the fact that the donor livers are flushed with preservation solution and therefore are almost free of blood and leucocytes.

## Supplementary Information

Below is the link to the electronic supplementary material.

Supplementary file1 (DOCX 4581 KB)

Supplementary file2 (DOCX 31 KB)

## Data Availability

The datasets supporting the conclusions of this article are included within the article and its supplementary files.

## Abbreviations

ALF: Acute liver failure
ATP: Adenosine triphosphate
CCC: Cholangiocellular carcinoma
cDNA: Complementary DNA
CLM: Colorectal liver metastasis
CT: Crossing threshold
CTx: Healthy donor allografts
DEGs: Differential expressed genes
DILI: Drug induced liver injury
DNA: Desoxyribonuclein acid
EAD: Early allograft dysfunction
ECD: Extended criteria donors
EDTA: Ethylene diamine tetraacetic acid
ER: Endoplasmatic reticulum
EtOH: Ethanol
FFPE: Formalin-fixed and paraffin-embedded tissue
H&E: Hematoxylin and eosin staining
HCC: Hepatocellular carcinoma
IR: Ischemia–reperfusion
MCD: Methionine/choline deficient diet
miRNA: Micro ribonucleic acid
MTx: Fatty liver allografts
MASLD: Metabolic Dysfunction-Associated Steatotic Liver Disease
MASH: Metabolic Dysfunction-Associated Steatohepatitis
OLT: Orthotopic liver transplantation
PNF: Primary non-function
qPCR: Quantitative polymerase chain reaction
RNA: Ribonucleic acid
Rpm: Rounds per minute
RT: Reverse transcription
TCA: Tricarboxylic acid
Y: Years
