# Supplementary material for: miRNA biomarkers to predict risk of primary non-function of fatty allografts and drug induced acute liver failures
Source: Mol Cell Biochem. 2024 Oct 18;480(4):2573–93. doi: 10.1007/s11010-024-05129-3 (PMC11961548; doi:10.1007/s11010-024-05129-3)
Supplement: Supplementary file 1 — Supplementary file1 (DOCX 4581 KB) [file 11010_2024_5129_MOESM1_ESM.docx]

# Supplementary Figures

**Supplementary Figure S1.** Lack of sex differences in fatty allograft associated PNFs.

**Supplementary Figure S2. (A)** Unchanged miRNAs in fatty allograft associated PNFs. **(B)** miRNAs not influenced by grades of hepatic steatosis in tissue of fatty allograft associated PNFs.

**A**

**B1**

**B2**

**B3**

**B4**

**B5**

**B6**

**B7**

**B8**

**B9**

**B10**

**Supplementary Figure S3.** Unresponsive miRNAs in liver tissue following organ storage and reperfusion injury.

**A**

**B**

**C**

**D**

**E**

**F**

**G**

**H**

**I**

**J**

**K**

**Supplementary Figure S4.** Unresponsive miRNAs in blood samples of OLT patients and reperfusion injury following liver resection.

**A**

**B**

**C**

**D**

**E**

**F**

**G**

**H**

**Supplementary Figure S5.** Regulation of PNF associated miRNAs in plasma. **(A)** The effects of warm ischemia following liver surgery. **(B)** The effects of cold ischemia and reperfusion injury following OLT (day 1-3).

**A1**

**A2**

**A3**

**B2**

**B1**

**Supplementary Figure S6.** miRNAs not influenced by grades of hepatic steatosis in plasma samples of successfully performed OLTs and liver resection cases. The patients are grouped into mild-moderate and marked steatosis, and we compared the regulation of miRNAs by considering the expression of day 0 and 1, as well as day 1 and 3.


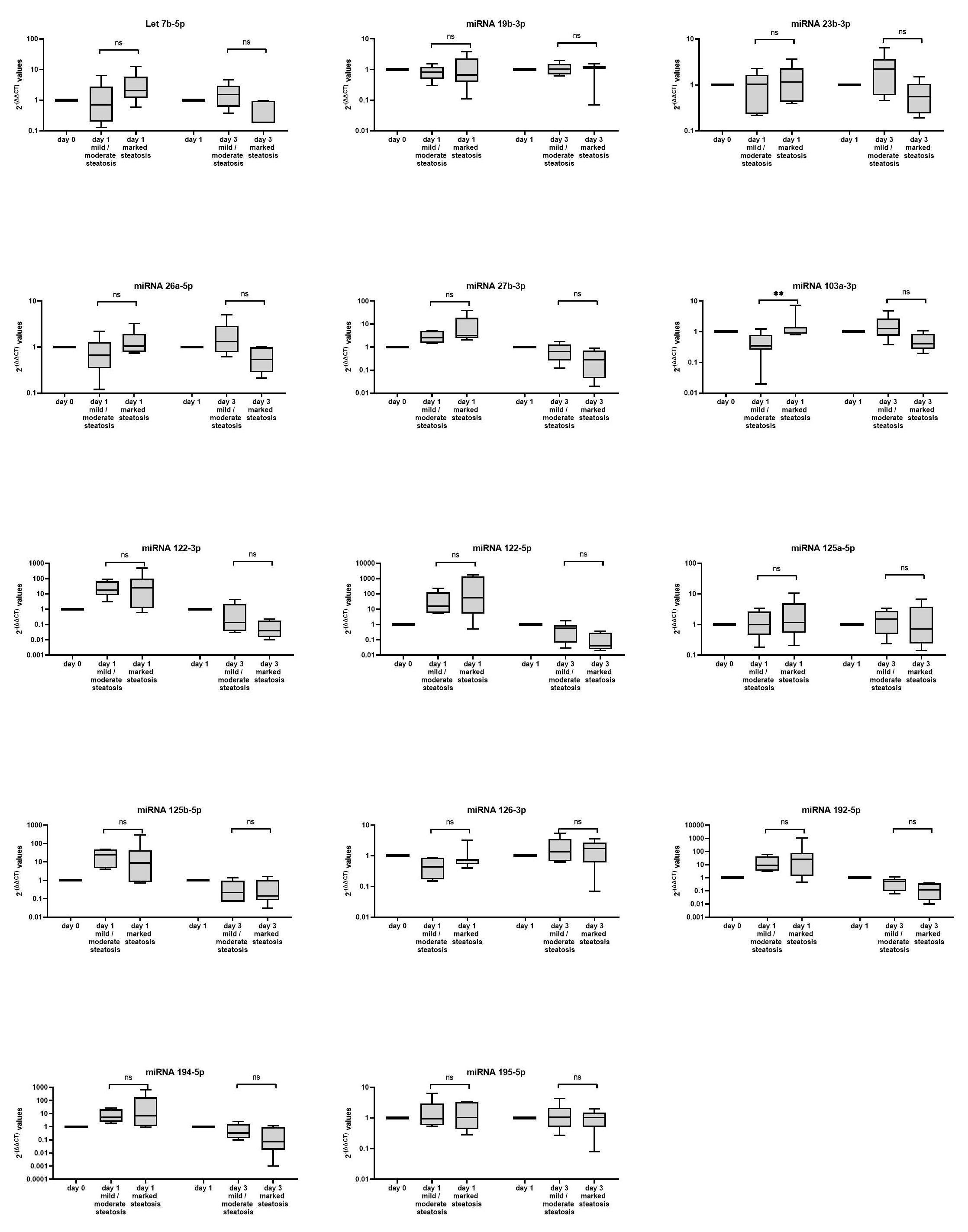


**A**

**B**

**C**

**D**

**E**

**F**

**G**

**H**

**I**

**J**

**K**

**L**

**M**

**N**
