# Supplementary material for: miRNA biomarkers to predict risk of primary non-function of fatty allografts and drug induced acute liver failures
Source: Mol Cell Biochem. 2024 Oct 18;480(4):2573–93. doi: 10.1007/s11010-024-05129-3 (PMC11961548; doi:10.1007/s11010-024-05129-3)
Supplement: Supplementary file 2 — Supplementary file2 (DOCX 31 KB) [file 11010_2024_5129_MOESM2_ESM.docx]

# Supplementary Tables

**Supplementary Table S1. (1A)** Patient characteristics. PNF-patients with marked hepatic steatosis. **(1B)** PNF-patients with mild-moderate hepatic steatosis. **(1C)** Patients with liver resections that served as controls.

**Supplementary Table S1A**

| **Patient ID** | **Gender** | **Age** | **Histology findings** |
| --- | --- | --- | --- |
| **Patient 1** | m | 43 | 40% macrovesicular steatosis |
| **Patient 2** | m | 30 | 80 % macro- and microvesicular steatosis, granulocytic inflammation |
| **Patient 3** | m | 29 | 60% macrovesicular steatosis |
| **Patient 5** | m | 49 | 15% macrovesicular steatosis |
| **Patient 6** | m | 62 | 80% macrovesicular steatosis |
| **Patient 7** | m | 48 | 40% macrovesicular steatosis, mixed inflammatory infiltrate |
| **Patient 8** | m | 63 | 20% macrovesicular steatosis, diffuse microvesicular steatosis, inflammatory infiltrate |
| **Patient 9** | m | 43 | 50% macrovesicular steatosis, periportal fibrosis, canicular cholestasis |
| **Patient 12** | m | 54 | 60% macro- and microvesicular steatosis, inhomogeneous lymphocytic and granulocytic inflammatory infiltrates |
| **Patient 13** | f | 57 | 60% macro- and 40% microvesicular steatosis, granulocyte infiltration, isolated endotheliitis |
| **Patient 16** | m | 36 | 10% macro and 60% microvesicular steatosis |
| **Patient 17** | f | 39 | 80% macrovesicular steatosis |
| **Patient 18** | f | 25 | 50% macrovesicular steatosis |
| **Patient 19** | f | 49 | 60% macrovesicular steatosis |
| **Patient 20** | m | 59 | 60% macrovesicular steatosis |
| **Patient 21** | f | 58 | 20% focal microvesicular steatosis |
| **Patient 23** | f | 48 | 30% macrovesicular steatosis |
| **Patient 24** | m | 50 | 30% macrovesicular and 50% microvesicular steatosis |
| **Patient 25** | m | 60 | 40% macrovesicular and 70% microvesicular steatosis, incipient granulocytic clearing reaction |
| **Patient 26** | m | 53 | 80% macro- and microvesicular steatosis, pericholangitis |
| **Patient 27** | m | 56 | 40% macrovesicular steatosis |
| **Patient 29** | f | 39 | 20% macrovesicular steatosis |

**Supplementary Table S1B**

| **Patient ID** | **Gender** | **Age** | **Histological grading of fatty allografts** |
| --- | --- | --- | --- |
| **Patient 4** | f | 54 | 10% macrovesicular steatosis, Inflammatory infiltrate of granulocytes in the portal fields |
| **Patient 10** | f | 56 | 10% macrovesicular steatosis, extensive strong microvesicular steatosis |
| **Patient 11** | m | 60 | 10% macrovesicular steatosis, lymphocytic and granulocytic inflammatory infiltrates |
| **Patient 14** | m | 35 | 10% macrovesicular steatosis |
| **Patient 15** | m | 57 | 10% macrovesicular steatosis |
| **Patient 22** | m | 20 | 10% macrovesicular steatosis, pericholangitis |
| **Patient 28** | m | 64 | 5% microvesicular steatosis |

**Supplementary Table S1C**

| **Patient ID** | **Gender** | **Age** | **Histology findings** |
| --- | --- | --- | --- |
| **Patient K1** | m | 75 | colorectal liver metastasis (CLM) |
| **Patient K2** | m | 69 | colorectal liver metastasis (CLM) |
| **Patient K3** | f | 48 | colorectal liver metastasis (CLM) |
| **Patient K4** | m | 64 | colorectal liver metastasis (CLM) |
| **Patient K5** | m | 70 | rectal liver metastasis (RLM) |
| **Patient K6** | m | 60 | colorectal liver metastasis (CLM) |
| **Patient K7** | m | 74 | colorectal liver metastasis (CLM) |
| **Patient K8** | m | 80 | colorectal liver metastasis (CLM) |
| **Patient K9** | f | 63 | Appendix carcinoma liver metastasis |
| **Patient K10** | m | 60 | NCC liver metastasis |
| **Patient K11** | m | 52 | Barett carcinoma liver metastasis |

**Supplementary Table S2.** Cycle conditions used for the qPCR.

| **Reaction type** | | **Time** | **Temperature** |
| --- | --- | --- | --- |
| Initial activation step | | 15 min | 95°C |
| Denaturation | 40x | 15 sec | 94°C |
| Annealing |  | 30 sec | 55°C |
| Extension |  | 30 sec | 70°C |

**Supplementary Table S3.** Primer sequences used for qPCR.

| **miScript primer assay** | **Targets mature miRNA** | **Sequence** |
| --- | --- | --- |
| Hs_miR-16_2 | hsa-miR-16-5p | 5'UAGCAGCACGUAAAUAUUGGCG |
| Hs_let-7b_1 | hsa-let-7b-5p | 5'UGAGGUAGUAGGUUGUGUGGUU |
| Hs_miR-19b_2 | hsa-miR-19b-3p | 5'UGUGCAAAUCCAUGCAAAACUGA |
| Hs_miR-23b_2 | hsa-miR-23b-3p | 5'AUCACAUUGCCAGGGAUUACC |
| Hs_miR-26a_2 | hsa-miR-26a-5p | 5'UUCAAGUAAUCCAGGAUAGGCU |
| Hs_miR-27b_2 | hsa-miR-27b-3p | 5'UUCACAGUGGCUAAGUUCUGC |
| Hs_miR-103a_1 | hsa-miR-103a-3p | 5'AGCAGCAUUGUACAGGGCUAUGA |
| Hs_miR-122*_1 | hsa-miR-122-3p | 5'AACGCCAUUAUCACACUAAAUA |
| Hs_miR-122a_1 | hsa-miR-122-5p | 5'UGGAGUGUGACAAUGGUGUUUG |
| Hs_miR-125a_1 | hsa-miR-125a-5p | 5'UCCCUGAGACCCUUUAACCUGUGA |
| Hs_miR-125b_1 | hsa-miR-125b-5p | 5'UCCCUGAGACCCUAACUUGUGA |
| Hs_miR-126_1 | hsa-miR-126-3p | 5'UCGUACCGUGAGUAAUAAUGCG |
| Hs_miR-192_1 | hsa-miR-192-5p | 5'CUGACCUAUGAAUUGACAGCC |
| Hs_miR-194_1 | hsa-miR-194-5p | 5'UGUAACAGCAACUCCAUGUGGA |
| Hs_miR-195_1 | hsa-miR-195-5p | 5'UAGCAGCACAGAAAUAUUGGC |
| Hs_miR-455-3p_1 | hsa-miR-455-3p | 5'GCAGUCCAUGGGCAUAUACAC |
| Ce_miR-39_1 | cel-miR-39-3p | 5'UCACCGGGUGUAAAUCAGCUUG |

**Supplementary Table S4.** Mean of the ∆CT ranges and the 95%-CI of the interpatient variability of PNF cases.

| miRNA | ∆CT range  Mean ± 95%-CI |
| --- | --- |
| *RNU 6B* | 1.81 ± 0.44 |
| *Let-7b-3p* | 1.43 ± 0.34 |
| *miRNA-19b-3p* | 1.09 ± 0.28 |
| *miRNA-23b-3p* | 0.93 ± 0.22 |
| *miRNA-26a-5p* | 1.17 ± 0.24 |
| *miRNA-27b-3p* | 0.73 ± 0.17 |
| *miRNA-103a-3p* | 1.6 ± 0.32 |
| *miRNA-122-3p* | 1.5 ± 0.31 |
| *miRNA-122-5p* | 1.04 ± 0.21 |
| *miRNA-125a-5p* | 0.86 ± 0.18 |
| *miRNA-125b-5p* | 0.97 ± 0.21 |
| *miRNA-126-3p* | 1.25 ± 0.25 |
| *miRNA-192-5p* | 1.57 ± 0.37 |
| *miRNA-194-5p* | 1.05 ± 0.22 |
| *miRNA-195-5p* | 1.39 ± 0.40 |
| *miRNA-455-3p* | 2.14 ± 0.78 |

**Supplementary Table S5.** Patient characteristics (**5A)** Donors of healthy liver allografts with T0 liver biopsies following cold ischemia. (**5B)** Patients with intraoperative biopsies following hilus occlusion during hepatectomy (warm ischemia).

**Supplementary Table S5A**

| **Patient ID** | **Gender** | **Age** | **Diagnosis / cause of death** | **Medical history** |
| --- | --- | --- | --- | --- |
| **Patient 1** | f | 53 | Subarachnoid hemorrhage (SH) | Smoking, no other comorbidities |
| **Patient 3** | m | 67 | Intracranial injury | NSEMI with stent, hypothyreosis, smoking |
| **Patient 6** | m | 51 | Subarachnoid hemorrhage | Aortic valve replacement and aorta ascendens replacement, hypertension |
| **Patient 9** | f | 67 | Subarachnoid hemorrhage | Hyperthyreosis, Myocardial infarction, hypertonic |
| **Patient 12** | f | 50 | Subarachnoid hemorrhage | Adiposities, smoking |
| **Patient 14** | f | 51 | Non traumatic intracranial hemorrhage | arterial hypertonic, T2-DM, PAVK, Kidney-insufficient, smoking |
| **Patient 17** | m | 50 | Subarachnoid hemorrhage | ASH, smoking (30 pack years) |

**Supplementary Table S5B**

| **Patient ID** | **Gender** | **Age** | **Histology** |
| --- | --- | --- | --- |
| **Patient 2** | m | 80 | CCC (Klatzkin) |
| **Patient 4** | f | 74 | CLM |
| **Patient 5** | m | 43 | CCC |
| **Patient 7** | m | 52 | CLM |
| **Patient 8** | f | 58 | CCC |
| **Patient 10** | f | 51 | CLM |
| **Patient 11** | f | 65 | CCC |
| **Patient 15** | m | 59 | CCC |
| **Patient 16** | m | 55 | CCC |
| **Patient 18** | m | 53 | CLM |

**Supplementary Table S6.** Patient characteristics of plasma samples.

| **Patient ID** | **Gender** | **Age** | **Diagnosis** |
| --- | --- | --- | --- |
| **Patient 1** | m | 55 | LTx |
| **Patient 3** | m | 63 | LTx |
| **Patient 6** | m | 52 | LTx |
| **Patient 7** | m | 52 | CLM |
| **Patient 8** | f | 58 | CCC |
| **Patient 9** | m | 61 | LTx |
| **Patient 10** | f | 51 | CLM |
| **Patient 11** | f | 65 | CCC |
| **Patient 12** | m | 49 | LTx |
| **Patient 14** | m | 58 | LTx |
| **Patient 15** | m | 59 | CCC |
| **Patient 16** | m | 55 | CCC |
| **Patient 17** | m | 67 | LTx |
| **Patient 18** | m | 53 | CLM |
